# A pilot study to examine COVID‐19 induced cognitive loss in diverse older populations

**DOI:** 10.1002/alz70860_107571

**Published:** 2025-12-23

**Authors:** Mahmood Mohamed, Shreya Upendra, Li Zhou, Ankit Parekh, Jessica Spat‐Lemus, Drew Melancon, Hiuyu Au, Yiyu Cao, Clara Li

**Affiliations:** ^1^ Icahn School of Medicine at Mount Sinai, New York, NY, USA; ^2^ Montclair State University, Montclair, NJ, USA

## Abstract

**Background:**

Cognitive loss following COVID‐19 has been recognized as an acute and long‐term sequela of the disease, with deficits in attention, executive functioning, and memory (Panagea et al., 2024; Hampshire et al., 2021). Older adults from racial and ethnic minority backgrounds face heightened risks of severe COVID‐19 outcomes due to aging‐related vulnerabilities and a higher prevalence of comorbidities, yet post‐infection cognitive impairment in these populations remains unexplored. Prior studies have been limited by suboptimal assessments, and recruitment of predominantly non‐Hispanic White adults. Our pilot study, therefore, aims to address this gap by examining the relationship between persistent COVID‐19 symptoms, and cognitive outcomes in a small sample of racially/ethnically diverse older adults.

**Method:**

20 participants were engaged from Mount Sinai's COVID‐19 Biobank (mean age 63.4 ± 4.9 years; 14.6 ± 3.9 years of education; 55% male; race/ethnicity: 45% Black, 15% Hispanic, 5% Asian, 35% non‐Hispanic White), and hospitalized in 2020. Inclusion criteria were: (1) History of SARS‐CoV‐2 infection or serum antibody positive (2) age ≥55, and (3) English‐speaking. Participants were remotely administered the NACC UDS 3.0 and several questionnaires, including one assessing persistent COVID‐19 symptoms. Univariate analyses were used to compare demographic characteristics between groups.

**Result:**

No group differences were found between participants with and without loss of smell/taste *during* infection. However, those with *long‐term* loss of smell/taste (>4 weeks) scored significantly lower on CERAD total, delay, and recognition (*p* = 0.016, 0.042). Additionally, longer hospitalization durations were significantly associated with poorer global cognition (*p* = 0.001), verbal memory (*p* = 0.014), auditory working memory (*p* = 0.043), simple attention and processing speed (*p* < 0.001), as well as verbal fluency (*p* = 0.009).

**Conclusion:**

Participants with *long‐term* loss of smell/taste and longer hospitalization had persistent cognitive impairment approximately 2.9 years post COVID‐19 infection. Therefore, findings suggest the importance of studying the long‐term role of infections similar to COVID‐19 on Alzheimer's Disease and Related‐Dementias (AD/ADRD) risk, particularly in a diverse sample of older populations. Further research with larger, longitudinal samples is needed to clarify the underlying mechanisms and inform cognitive rehabilitation strategies.

